# Preparation and Characterization of Chitosan Films Containing Lychee (*Litchi chinensis* Sonn.) Pericarp Powder and Their Application as Active Food Packaging

**DOI:** 10.3390/foods10112834

**Published:** 2021-11-17

**Authors:** Longwei Jiang, Zhao Luo, Haibi Liu, Fenghui Wang, Hanyu Li, Hechen Gao, Huajiang Zhang

**Affiliations:** 1 College of Engineering, Northeast Agricultural University, Harbin 150030, China; jianglw@neau.edu.cn (L.J.); liu13654558545@163.com (H.L.); G1099287155@163.com (H.G.); 2 College of Food Science, Northeast Agricultural University, Harbin 150030, China; a13329648783@163.com (Z.L.); fhuiwang@163.com (F.W.); lihanyu1004@126.com (H.L.)

**Keywords:** active packaging, chitosan, lychee pericarp, antimicrobial ability, antioxidant ability, food preservation

## Abstract

In this study, lychee (*Litchi chinensis* Sonn.) pericarp powder was added to chitosan (CHS) matrix to develop active packaging films, and their structure, physicochemical, antibacterial, antioxidant, and functional properties were investigated. FT-IR results showed that intermolecular hydrogen bonds were formed between CHS and polyphenols in lychee pericarp powder (LPP), and the intermolecular interaction interfered with the assembly of CHS into semi-crystal structure, which reduced the crystallinity of CHS film. Incorporation of LPP significantly reduced water vapor permeability, water solubility, swelling degree, and elongation at break of CHS film (*p* < 0.05). However, UV-visible light barrier, tensile strength, and antibacterial and antioxidant properties of CHS films were increased by LPP incorporation. CHS-LPP film remarkably lowered the weight loss, firmness, titratable acidity, and total soluble solids of fresh-cut apple after five days storage. CHS-LPP film packaging effectively inhibited the browning of fresh-cut apple and the reduction of polyphenol content in apple juice caused by polyphenol oxidase (PPO)-mediated oxidation during storage. Therefore, CHS-LPP films have great potential as food packaging material to ensure the quality and extend the shelf life of food products.

## 1. Introduction

Active packaging refers to an innovative packaging system that surpasses the original functions of traditional packaging systems [1]. Active packaging enables the food products, the packaging materials, and the environment interact in a positive way to improve sensory properties and food safety while maintaining food quality to extend the shelf life of food [2]. Generally, this extended shelf life is achieved by adding active materials, such as antioxidant and antibacterial compounds, inside or on the surface of packaging materials [3,4]. Different active substances in food packaging materials have different action mechanisms depending on active substance nature, food matrix, and polymer materials [5,6].

Most of the materials used in food packaging industry are synthetic polymers obtained from petrochemical products, which cause serious environmental problems due to their non-degradability [7]. In the field of active packaging, biodegradable and natural biopolymers (such as polysaccharides and proteins) have attracted more and more attention [8,9]. Among all kinds of biopolymers, chitosan (CHS), a natural polysaccharide, is widely used to prepare food packaging film due to its excellent film-forming property and low cost [10]. CHS is commercially produced from shellfish processing waste and has a unique, positively charged biopolymer. Additionally, CHS film can also be combined with natural antibacterial agents and antioxidants, such as polyphenols, essential oils, and agricultural wastes, to obtain biological activity [11,12,13].

Lychee (*Litchi chinensis* Sonn.) is a popular specialty fruit grown commercially in subtropical and tropical regions [14]. It has become one of the most popular fruits for consumers due to its attractive red peel and appealing taste, with annual production of about 1.5 million tons in China [15,16]. Lychee is usually eaten as processed fruit or fresh fruit. The consumption of lychee produces a great deal of waste, especially the pericarp, which weighs as much as 15% of the fresh weight of the entire lychee [15]. The pericarp is usually burned or discarded directly, so further utilization of these by-products may help to reduce environmental problems and waste [4]. Lychee pericarp contains large amounts of procyanidins, which are polyphenols composed of different numbers of flavan-3-ol subunits, such as gallocatechin, epicatechin, and catechin [15,17]. Moreover, researches have reported that procyanidins have a variety of effects, such as antibacterial, antioxidant, anti-aging, and anti-cancer [17,18,19,20]. Nowadays, the research on lychee pericarp mainly focuses on the extraction of active ingredients [15,21]. However, only a few studies have focused on the development of active packaging materials based on lychee pericarp extract. For example, Liu et al. [22] added nano-titanium dioxide and lychee pericarp extract to the CHS matrix to develop a new novel active packaging film. It is worth noting that extraction is a time- and energy-consuming process. In addition, the residue of extraction solvent and the instability of the extract limit the application of lychee pericarp extract in food packaging [13]. In contrast, it is more economical and convenient to develop active packaging by directly adding lychee pericarp powder (LPP), which can also achieve the same antibacterial and antioxidant effects [1]. Compared with the extract, the fruit peel powder usually has low solubility, and the formed active film has granular texture, which may affect the physical appearance of the packaging [13]. Many scholars use new strategies to develop active packaging materials by combining fruit peel as functional fillers with biopolymers, like those based on passion fruit peel powder [23], papaya peel powder [24], prickly pear peel powder [25], pomegranate peel powder [1], mango peel powder [26], and mangosteen peel powder [13], among others. However, as far as we know, there are no specific studies just focusing on the combination of LPP with CHS to form active packaging. There are still obvious gaps in understanding the structure and properties of CHS-LPP film.

Apple is one of the most popularly produced fruits in the world. It is widely eaten due to its flavor, firmness, appearance, and nutritional characteristics [27]. The nutritional characteristic is becoming more and more important because apple is an important source of health-promoting compounds. Several epidemiological studies have reported that these compounds can protect health [28]. As the trend of apple consumers is changing, apples are often processed into fresh-cut apple and apple juice. However, fresh-cut apple is difficult to preserve because the cutting operation increases microbial growth, softening, and respiration rate, and apple juice is vulnerable to oxidation when exposed to air; therefore, the application of minimal processing for their preservation is increasing [26,29]. Rojas Bravo et al. [26] developed a mango peel powder/starch coating to preserve apple slices, which is a very effective way for the preservation of fresh-cut apple. However, fresh-cut apple with a coating may lose its natural taste. In addition, the preservation method of active coating is only applicable to solid food, which limits its application in food preservation. Active packaging film may be a more effective way to preserve food, whether for solid food or beverage. Therefore, this study LPP rich in polyphenols was directly incorporated into CHS film matrix for the first time to develop an active packaging system. The aim of this study was to investigate the effects of LPP content on the structure, physicochemical, antioxidant, and antibacterial properties of CHS-LPP films and assess their effect as active packaging on the quality of fresh-cut apple and apple juice during storage.

## 2. Materials and Methods

### 2.1. Materials

Lychee (*Lichi chinensis* Sonn. Cv. Guiwei) and red Fuji (*Malus pumila mill*) apples were obtained from the local market (Harbin, China). CHS (viscosity of 100–200 mpa∙s and deacetylation degree of 95%), Folin–Ciocalteu reagent and 2,2′-Azino-bis (3-ethylbenzothiazoline-6-sulfonic acid) diammonium salt (ABTS) were purchased from Macklin Biochemical Co., Ltd. (Shanghai, China). Glycerol was purchased from Tian in Fuyu Fine Chemical Co., Ltd. (Tianjin, China). 1,1-diphenyl-2-picrylhydrazyl (DPPH) was obtained from Shanghai yuanye Bio-Technology Co., Ltd. (Shanghai, China). *Escherichia coli* and *Staphylococcus aureus* were obtained from Beijing Microbiological Culture Collection Center (Beijing, China).

### 2.2. Preparation and Characterization of LPP

The lychee was cleaned and peeled, and the lychee pericarp was torn into small pieces, then frozen in liquid nitrogen and freeze-dried. After that, the lychee pericarp was ground into powder using ultramicro plant crusher (FZ102, Taisite, Tianjin, China) and passed through 80-mesh sieve to obtain LPP. The separated particles were smaller than 180 μm (80 mesh). The morphology of the particles was observed by field emission scanning electron microscope (SU8010, Hitachi, Tokyo, Japan). The content of total phenols in LPP was determined by Folin–Ciocalteu method [30].

### 2.3. Film Preparation

CHS (2 g) was dissolved in 100 mL of 2% acetic acid solution (*v*/*v*) at 50 °C with stirring of 800 rpm for 1 h. Then 30 wt% (based on CHS weight) glycerol was added and stirred continuously for 10 min. After that, LPP with different contents (0, 2.5, 5, 7.5, and 10 wt%) on CHS basis was added into the film solutions and stirred continuously for 30 min. The final film-forming solution was degassed and cast onto a plexiglass mold (20 cm × 20 cm) and dried at room temperature for 2 days. All the films were equilibrated at 75% relative humidity for 48 h before testing [31]. The films containing different contents of LPP (from 2.5 to 10 wt%) were named as CHS-2.5LPP, CHS-5LPP, CHS-7.5LPP, and CHS-10LPP.

### 2.4. Characterization of Films

#### 2.4.1. Structural Characterization of the Films

Field emission scanning electron microscope (SEM) (Hitachi S-3400 N, Tokyo, Japan) was used to observe the surface and cross-sectional morphology of the film. ATR (attenuated total reflectance) Fourier-transform infrared (FT-IR) spectra of the film was obtained using a spectrometer (Nicolet is50, Thermo Fisher Scientific, Waltham, MA, USA) in range of 4000–400 cm^−1^. X-ray diffraction (XRD) pattern was analyzed using an X-ray diffractometer (D8 Advance, Bruker, Karlsruhe, Germany) with Cu Kα radiation between 2θ = 5° and 50°.

#### 2.4.2. Optical Properties of Films

The physical appearance of the film was recorded by a film sample covered on printed paper with school logo. The color of the film samples were measured using a colorimeter (CR-20, Konica Minolta, Tokyo, Japan) with a white plate (L* (94.90), a* (−0.2), and b* (4.3)) as the standard background. The film samples were evenly distributed on a standard white reflector plate as a background, and the color parameters (*a*, *b*, and *L*) were determined [1]. The total color difference (Δ*E*) between the CHS-LPP films and CHS film was calculated as follows:

(1) $$∆E=\sqrt{{\left(L^{*}-L\right)}^{2}+{\left(a^{*}-a\right)}^{2}+{\left(b^{*}-b\right)}^{2}}$$

where L* (87.47), a* (−2.5), and b* (0.13) were the color parameters of CHS film control that was used for comparison.

UV-Vis spectrophotometer (UV-2600, Shimadzu, Kyoto, Japan) was used to measure the light transmittance of the film sample between 200 and 800 nm.

#### 2.4.3. Thickness, Moisture Content (MC), Water Solubility (WS), and Swelling Degree (SD)

The thickness of the film was determined by a helical micrometer (Harbin Measuring & Cutting Tool Group Co., Ltd., Harbin, China). The MC and SD of the film were measured using the previous method [32]. The film samples were dried to constant weight at 105 °C, and the MC of the film was calculated by the weight loss of the film. The film (40 mm × 10 mm) was immersed in distilled water (30 mL) for 24 h, and the SD was determined by the weight of the film after absorbing water relative to its initial weight. The WS of the film was measured using the method of Wang et al. with slight modification [33]. The film sample dried to constant weight was placed in 100 mL distilled water and stirred with 150 rpm for 6 h. The WS was the ratio of the dry matter of the film dissolved in water.

#### 2.4.4. Water Vapor Permeability (WVP)

The WVP of the film were measured using the previous method with slight modifications [32]. A special aluminum cup with inner diameter of 6.8 cm, outer diameter of 8.0 cm (exposed area of 36.3 cm^2^), and depth of 5.5 cm was used. The film sample was sealed over the cup containing 6 g anhydrous CaCl_2_ and placed into a desiccator with RH of 95% at room temperature (25 °C) [31,32]. After reaching the steady-state condition (about 2 h), the cup was weighed every 2 h for 20 h. Changes in the weight of the cup were recorded using an analytical balance (Mettler-Toledo Instrument (Shanghai) Co. Ltd., Shanghai, China) and plotted as a function of time. The slope of the linear portion of this plot represented the amount of water vapor permeating through the film per unit time. Steady state over time (slope) yielded a regression coefficient of 0.99 or greater. The water vapor transmission rate (*WVTR*, g·m^−^^2^·h^−^^1^) was determined from the slope (g·h^−^^1^) divided by the exposed area (36.3 cm^2^). WVPR values were corrected to account for resistance of the stagnant air gap (1 cm) between the underside of film samples mounted on cups and the surface of desiccant inside cups [34]. Then, WVP was calculated using the following equation:

(2) $$\mathrm{WVP}\;\left({g\cdot m}^{-1}\cdot h^{-1}{\cdot \mathrm{Pa}}^{-1}\right)=\frac{WVTR\times d}{p\times \left(R_{1}-R_{2}\right)}$$

where *d* is the thickness of the film (m), *p* is the saturation vapor pressure of water at test temperature (25 °C), *R*_1_ is the relative humidity in the desiccator, and *R*_2_ is the relative humidity inside the cup.

#### 2.4.5. Mechanical Properties

Mechanical properties, including elongation at break (EAB), tensile strength (TS), and elastic modulus (EM), of the film (1 cm × 6 cm) were determined by tensile-testing machine (Model QJ 210, Shanghai Qingji Instrument Technology Co., Ltd., Shanghai, China) based on our previous research [32]. The 100 N sensor was selected, and the tensile speed was set to 10 mm/min.

### 2.5. Antimicrobial Properties

The disc diffusion method was used to evaluate the antibacterial properties of the film [1]. *Escherichia coli* and *Staphylococcus aureus* were inoculated in the nutrient broth and activated at 30 °C for 24 h before the experiment. The sample (6 mm in diameter) was laid on the lysogeny broth agar plate and then incubated at 37 °C for 24 h. After that, the widths of the inhibition zones were measured.

### 2.6. Antioxidant Properties

The DPPH radical scavenging activity of the film was measured according to the previous study with slight modification [32]. The film (20 mg) was put into test tube and immersed into 0.2 mM DPPH ethanol solution (4 mL) for 30 min in the dark. Then, the absorbance was determined at 517 nm. The DPPH radical scavenging activity was calculated using the following equation:

(3) $$\mathrm{DPPH}\;\mathrm{radical}\;\mathrm{scavenging}\;\left(\%\right)=\frac{A_{control}-A_{sample}}{A_{control}}\times 100$$

where *A_sample_* is the absorbance of the test film, and *A_control_* is the absorbance of the control without film.

The total antioxidant activity of the film was evaluated by ABTS method [29]. First, ABTS solution (2 mL, 7.4 mM) and potassium persulfate solution (2 mL, 2.6 mM) were mixed in a centrifuge tube, and the solution was placed in dark for 12–16 h. Then, the mixture was diluted with ethanol until the absorbance at 734 nm was 0.7 ± 0.02. The film (20 mg) was added into diluted solution (4 mL) and allowed to stand in dark for 6 min. Then, the absorbance of the solution was measured at 732 nm. The total antioxidant activity of the film was calculated using the following equation:

(4) $$\mathrm{Total}\;\mathrm{antioxidant}\;\mathrm{activity}\;\left(\%\right)=\frac{A_{control}-A_{sample}}{A_{control}}\times 100$$

where *A_sample_* is the absorbance of the test film, and *A_control_* is the absorbance of the control without film.

### 2.7. Application as Fresh-Cut Apple Packaging

The fresh apple was cut into cubes (1 cm^3^). Three small cubes were heat-sealed into a 4 cm × 4 cm film, and then, the film pocket was put into an uncovered petri dish and stored in a desiccator (outer diameter 467 mm; height 465 mm) with 50% relative humidity for 5 days at room temperature (25 °C). Each desiccator contains three groups of parallel samples, and the samples were transferred to a new desiccator every day to ensure that the air composition is not significant altered by fruit respiration rate. The appearance of apples before and after storage was compared.

#### 2.7.1. Weight Loss and Firmness

The weight loss of the fresh-cut apple was calculated using the following equation:

(5) $$\mathrm{Weight}\;\mathrm{loss}\;\left(\%\right)=\frac{W_{0}-W_{n}}{W_{0}}\times 100$$

where *W*_0_ represents the initial weight of fresh-cut apple, and *W_n_* represents the weight of fresh-cut apple at nth days.

The firmness was determined by texture analyzer (TA-XTplus, Stable Micro Systems, London, UK), equipped with a cylindrical probe (diameter 5 mm). The operating conditions were set as 1 mm/s and 30% compression. Six fresh-cut apple blocks were measured in each group.

#### 2.7.2. Total Soluble Solids and Titratable Acidity

Fresh-cut apples were homogenized, mixed, and filtered, and then, the total soluble solids content was measured using a refractometer (LH-Q32, Luheng Biotechnology Co., Ltd., Hangzhou, China).

The titratable acidity was determined by titration method [35]. The ground, fresh-cut apple (5 g) was mixed with distilled water (50 mL) and then filtered with absorbent cotton. One to two drops of phenolphthalein were added to the filtrate (10 mL) as an indicator and titrated with NaOH (0.1 mol/L) until the endpoint was pink (pH 8.1). Titratable acidity was expressed as the grams of malic acid per 100 g of fresh-cut apple mass.

### 2.8. Application for Preserving the Apple Juice

Fresh apples were washed, peeled, weighed, and then made into apple juice with ten times the quality of ultra-pure water. The apple juice (10 mL) was heat-sealed into a 5 cm × 5 cm film, and the film pocket was placed at room temperature (25 °C) for testing. The measurements were carried out at 0, 12, and 24 h. Folin–Ciocalteu method was used to determine the content of total phenolic in apple juice [29]. Folin–Ciocalteu reagent (200 μL) was added to the test tube containing 1 mL apple juice sample. The mixture was shaken and allowed to rest for 3 min. After that, 3 mL of 2% (*w*/*v*) Na_2_CO_3_ solution was quickly added, shaken well, and stored at room temperature for 30 min. Then, the absorbance of the solution was measured at 760 nm and compared to a gallic acid (GA) calibration curve. The result was expressed in mg GA/mL apple juice.

### 2.9. Statistical Analysis

All tests were repeated at least three times, and the results were expressed as mean ± standard deviation. Data were analyzed by Duncan’s test with SPSS software, and *p* < 0.05 was statistically significant.

## 3. Results and Discussion

### 3.1. Characterization of LPP

**Figure 1:**
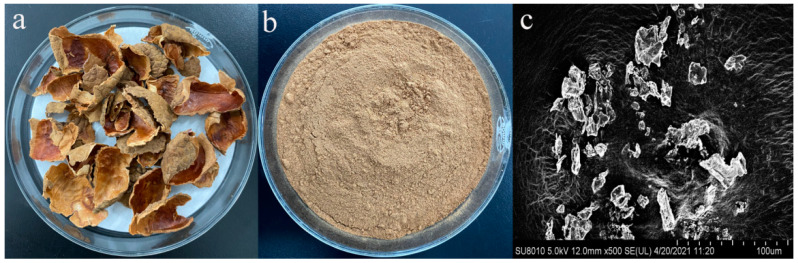
Freeze-dried lychee pericarp (**a**), LPP (**b**), and SEM image of LPP (**c**).

Freeze-dried lychee pericarp, LPP, and SEM image of LPP are shown in Figure 1. Lychee pericarp presented brown color after freeze-drying (Figure 1a). Then, the lychee pericarp was ground and sieved to obtain LPP (Figure 1b). The SEM results further showed that the LPP presented block structure (Figure 1c). The total phenol content in LPP was 44.1 mg GA equivalent/g powder. The potential phenolic compounds in LPP were epicatechin, catechin, procyanidins, flavonoids and phenolic acids [22].

### 3.2. Characterization of Films

#### 3.2.1. Structural Characterization of the Films

**Figure 2:**
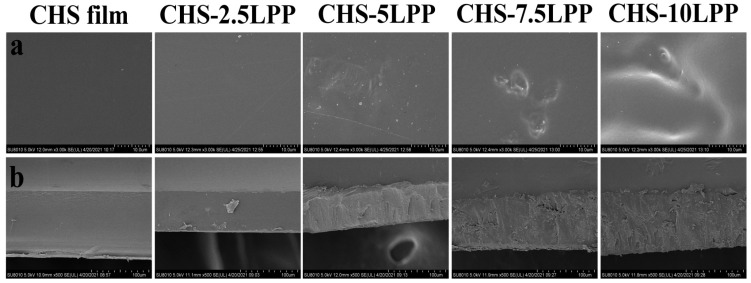
SEM images of the surface (**a**) and cross-section (**b**) of the films.

SEM images of the surface and cross-section of the films are shown in Figure 2. The microstructure of CHS film was smooth and uniform. The same result was also observed by other researchers [13]. It can be observed that the roughness of the film surface increases after adding LPP. de Moraes Crizel et al. [24] also observed rough surfaces in papaya peel powder-gelatin films due to the formation of powder clusters in gelatin matrix. Although the surface roughness of the films increased, the surface structure of the films was still dense. The cross-section of CHS film and CHS-2.5LPP were uniform and smooth, and a small amount of LPP particles can be observed in the cross-section of CHS-2.5LPP, which indicates that CHS, glycerol, and low concentration LPP (2.5 wt%) have good phase compatibility. However, with the increase of LPP content, the roughness of the cross-section of CHS-LPP films increases gradually, and the cross-section becomes uneven. The reason was that LPP was embedded into the film matrix, which makes the internal structure of the film discontinuous [13]. This finding was consistent with that of Hanani et al. [1], who observed a similar phenomenon due to the addition of pomegranate peel in gelatin film, resulting in structural discontinuity and reducing the EAB of the film.

**Figure 3:**
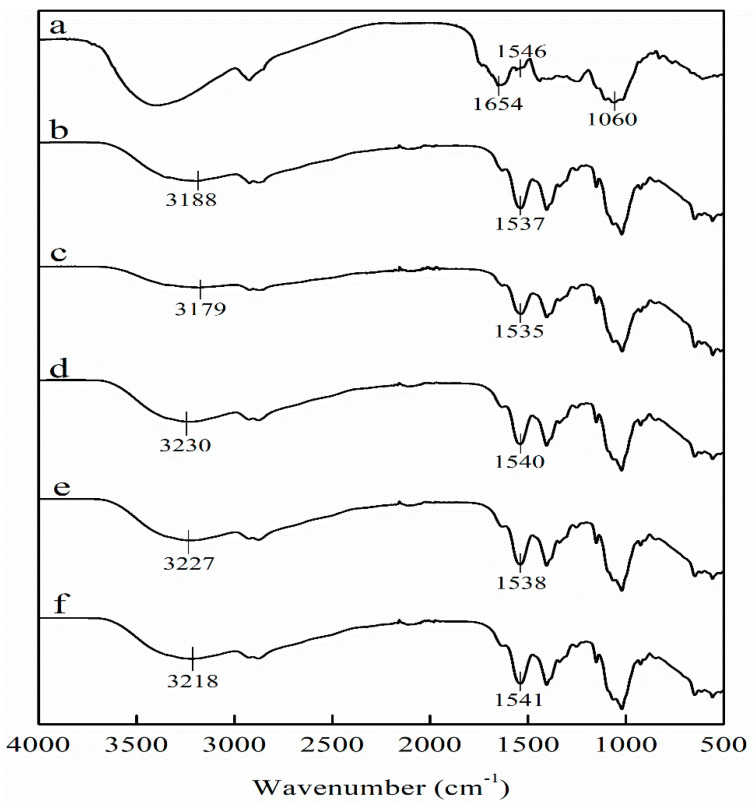
FT-IR spectra of LPP (**a**), CHS film (**b**), CHS-2.5LPP (**c**), CHS-5LPP (**d**), CHS-7.5LPP (**e**), and CHS-10LPP (**f**).

The FT-IR spectra of the LPP and films are shown in Figure 3. The spectra for LPP shows characteristic bands of phenolic compounds at 1654, 1546, and 1060 cm^−1^ corresponded to C=O stretching vibration, C=C stretching of aromatic ring, and C-O deformation of aromatic ring [13]. In FT-IR spectra of CHS film, the broad band at about 3188 cm^−1^ represented stretching vibration of N-H and O-H. The characteristic bands at 1346, 1407, 1537, and 1631 cm^−1^ corresponded to C-N stretching, C-H bending, N-H bending, and C-O stretching of amide group, respectively [36]. The addition of LPP did not significantly change the FT-IR spectra of CHS films, while the FTIR spectra of CHS-LPP films had similar profiles. Zhang et al. [37] also found that the FTIR spectra of the CHS film did not change significantly after adding mangosteen rind powder, confirming that mangosteen rind powder and the CHS matrix have good compatibility. However, after adding LPP to the CHS film, the positions of the peaks at 3188 and 1537 cm^−1^ shifted. The changes in the peak position were due to the formation of intermolecular hydrogen bonding interactions between the hydroxyl/amino groups of CHS and the hydroxyl groups of polyphenols [13].

**Figure 4:**
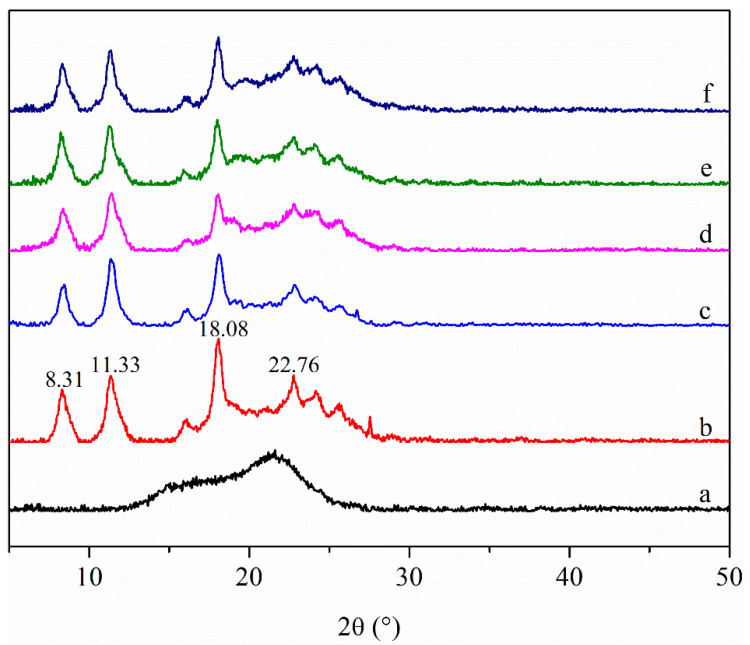
XRD patterns of LPP (**a**), CHS film (**b**), CHS-2.5LPP (**c**), CHS-5LPP (**d**), CHS-7.5LPP (**e**), and CHS-10LPP (**f**).

The XRD patterns of the films and LPP are shown in Figure 4. The XRD pattern of LPP has a broad peak around 22°, indicating that LPP was in an amorphous state. Different from LPP, CHS film exhibited a semi-crystalline state with four obvious diffraction peaks at 2θ = 8.31, 11.33, 18.08, and 22.76°, which correspond to the crystal plane of (200), (201), (202), and (311), respectively [13,36]. The peaks at 8.31 and 11.33° were the hydrated crystallite structure formed by the integration of water molecules in the lattice, and the peak at 18.08° was attributed to the regular lattice of CHS [38]. After adding LPP to the CHS film, the intensity of the diffraction peaks and the crystallinity of the films were reduced, and the position of the diffraction peak was slightly shifted. This was due to the formation of intermolecular hydrogen bond interaction between LPP and CHS, which interferes with the assembly of CHS into semi-crystal structure [36].

#### 3.2.2. Optical Properties of Films

**Figure 5:**
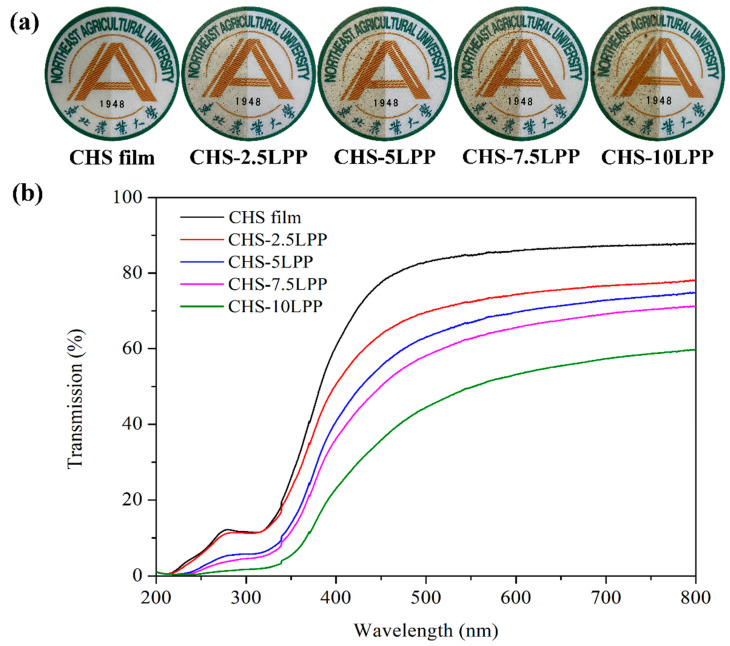
The physical appearance (**a**) and UV–vis light transmittance (**b**) of the films.

The physical appearance of the films is shown in Figure 5a. CHS film was almost colorless and transparent, but CHS-LPP showed yellow color, and the color gradually became dark with the increase of LPP content. In addition, LPP particles were also observed in CHS-LPP. Table 1 shows the color parameters of the film, *a* (green to red), *b* (blue to yellow), *L* (lightness), and Δ*E* (total color difference). With the increase of LPP content, the *L* value decreased significantly (*p* < 0.05), indicating that the lightness of the film became dark. Hanani et al. [1] also found that the lightness of fish skin gelatin film decreased when pomegranate peel powder was incorporated into the film. The decrease of film lightness helps to prevent the oxidation deterioration of packaged food due to exposure to visible light [1]. With the increase of LPP content, the *a* value of the films increased significantly (*p* < 0.05), indicating that the red color of the films increased. The reason was the presence of anthocyanins in LPP. Anthocyanins are a group of polyphenols that are the purple, orange, and red pigments of flowers, fruits, and various plants [39]. The b value increased significantly (*p* < 0.05), indicating that as the LPP content increased, the yellowness of the film increased. Zhang et al. [37] also found a similar phenomenon when the addition of mangosteen rind powder increased the yellowness of the CHS film. The Δ*E* of CHS films increased significantly with increasing LPP levels compared to the control film (*p* < 0.05), which indicated that LPP had a high effect on the color of films due to its intrinsic brown color. Notably, the color of CHS-LPP films gradually deepened with the increase of LPP content, which was reflected by the gradually increased Δ*E* of these films.

Figure 5b shows the light transmittance of the films. The CHS film exhibits the highest light transmittance, which was due to the lack of UV-vis absorbing groups [40]. However, with the increase of LPP content, the light transmittance of CHS-LPP films decreased gradually. There are two main reasons for this phenomenon. First, polyphenols in LPP have strong absorption ability to UV-Vis radiation. Secondly, the LPP particles dispersed in the film matrix can scatter or block the propagation of light [41]. Especially, with the increase of LPP content, the light transmittance of CHS-10LPP at 200–300 nm was close to zero, while the UV transmittance of pure CHS film was still more than 10%, which indicates that the film has strong UV light absorption ability. This is beneficial to inhibit UV-induced oxidation in food systems. In general, food products need to be stored away from light. The reduction of the light transmittance of the film can reduce the influence of light on food, which was conducive to the storage of food products [32]. It is worth noting that food is usually not directly exposed to the UV light region unless it is sterilized by UV radiation. Therefore, the food packaged with CHS-LPP film is not suitable for UV sterilization, and it is best to treat the food before packaging.

#### 3.2.3. Thickness, MC, WS, and SD

The thickness, MC, WS, and SD of films are shown in Table 2. The thickness of CHS film increased significantly after LPP was added (*p* < 0.05) because of the increase of solid content in the film. The MC of CHS film was the highest because of the strong intermolecular hydrogen bond between CHS and water [13]. However, the MC of CHS-LPP films were relatively lower because the interaction between LPP and CHS limits the formation of hydrogen bonds between water and CHS [22]. Lychee pericarp contains hydrophobic compounds, such as catechin, rutin, and quercetin, which contribute to the hydrophobicity of LPP [22]. Therefore, the decrease of MC in CHS-LPP films was due to the hydrophobicity of LPP, which indicates that the affinity of CHS-LPP films to water was low. With the increase of LPP content, WS and SD of CHS films decreased significantly (*p* < 0.05). The decrease of WS and SD was due to the hydrophobicity of CHS-LPP films. In addition, the strong interaction between CHS and LPP reduces the swelling rate and water absorption of the polymer chains located in the interface region [32].

#### 3.2.4. WVP

The WVP of the films is shown in Table 3. The WVP of CHS film was 6.35 × 10^−7^ g·m^−1^·h^−1^·Pa^−1^. After adding LPP, the WVP of CHS-LPP films decreased significantly (*p* < 0.05). The low WVP of CHS-LPP films may be related to the interaction of hydroxyl and amino groups in CHS with carboxyl and hydroxyl groups of phenolic compounds in LPP, which can narrow the passage of water molecules [37]. Due to the hydrophobicity of LPP, the addition of LPP increased the hydrophobicity of the films and reduced water absorption. Moreover, the added LPP could enter into the free volume of the films that obstructed the passage of water vapor [36]. Liu et al. [22] also found that the WVP of CHS film containing litchi peel extract was lower than pure CHS film. Moro et al. [23] reported a decrease in WVP when combining passion fruit peel with starch due to the introduction of tortuous paths for water molecules to pass through the presence of small particles present in the matrix. In addition, FT-IR results showed that intermolecular hydrogen bonds were formed between LPP and CHS, which reduced the effectiveness of hydrophilic groups in the film, thus limiting the affinity of the film to water molecules [32].

#### 3.2.5. Mechanical Properties

**Figure 6:**
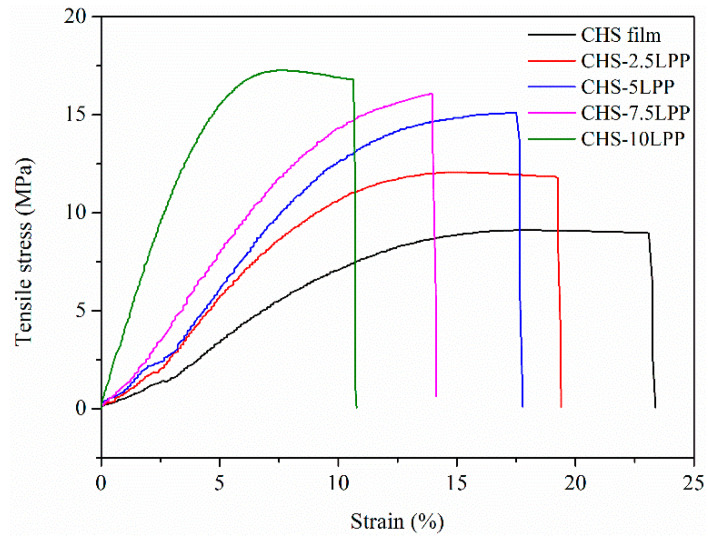
Stress-strain curves of the films.

The stress-strain curves obtained from tensile measurements of CHS and CHS-LPP films are shown in Figure 6, while the TS, EAB, and EM of the films were included in Table 3. The addition of LPP resulted in the progressive modification of the stress-strain profiles of CHS films. After adding LPP, the tensile stress of the CHS film increases, associated with a remarkable decrease of the strain, demonstrating a brittleness behavior [42]. With the increase of LPP content, the TS of CHS film increased significantly (*p* < 0.05). The enhancement of TS of the film was due to the interaction between CHS and LPP, which produces strong interfacial bonding force [43]. In addition, LPP can be used as reinforcement filler to strengthen the film network, which can effectively resist mechanical stress. On the contrary, the addition of LPP significantly reduced the EAB of CHS film (*p* < 0.05). The reason was that the structure of the film was discontinuous due to the addition of LPP in CHS, and the insoluble part of LPP destroys the structure of the film [13]. Hanani et al. [1] also found that after adding pomegranate peel powder to gelatin film, TS increased, and EAB decreased. The decrease in EAB leads to a decrease in the flexibility of the film, which increases the difficulty of industrial processing, such as processing the film into food preservation roll film. Therefore, improving the EAB of CHS-LPP film is worthy of further study. In addition, an increase of EM values was noticed in CHS-LPP films, indicating a higher rigidity due to the decrease of free volume [44]. This behavior can be related to the interactions among CHS and LPP, as shown by FT-IR analysis, which would lead to increasing resistance and rigidity but decreasing flexibility [45].

### 3.3. Antimicrobial Properties

The antimicrobial ability of CHS films is shown in Table 4. The antimicrobial ability of CHS was due to the cationic property of CHS, which can interact with the cell membrane of food borne pathogens and increase the cell permeability [13]. The antimicrobial activity of CHS-LPP films was higher than that of CHS, and the antimicrobial activity of CHS-LPP film was significantly enhanced with the increase of LPP content (*p* < 0.05). This could be attributed to the fact that the LPP was rich in polyphenols, and many studies have shown that polyphenols have antibacterial activity [46]. Polyphenols can inhibit bacteria by increasing the permeability of cell membrane, cell deformation, and inhibiting the synthesis of DNA or RNA [13]. Other researchers have also reported that adding pomegranate peel powder to fish gelatin film can enhance the antimicrobial activity [1]. In addition, the inhibition of CHS and CHS-LPP films on gram-negative bacteria (*Escherichia coli*) was weaker than that on gram-positive bacteria (*Staphylococcus aureus*). These different antimicrobial abilities can be explained in two ways: (i) the antimicrobial effect of CHS depends on the nature of bacteria, and there are differences in cell wall structure, cell physiology, and metabolism between gram-positive and gram-negative bacteria [13]; (ii) a study has reported that polyphenols are more effective against gram-positive organisms [47]. The synergistic effect may be increased when CHS and polyphenol-rich LPP were added together due to the CHS-polyphenol interactions. Therefore, the inhibitory effect of the films on gram-positive bacteria was increased. A study carried out by Zhang et al. [13] also showed that the antimicrobial activity of mangosteen rind powder/CHS film was stronger against gram-positive bacteria than against gram-negative bacteria. Our results suggested the developed CHS-LPP films could be further used to inhibit food spoilage caused by bacterial contamination.

### 3.4. Antioxidant Properties

The DPPH radical scavenging activity and the total antioxidant activity of the films are shown in Table 4. The DPPH radical scavenging activity and total antioxidant activity of the films were significantly improved with the increase of LPP content (*p* < 0.05). When the LPP content was 10%, the DPPH radical scavenging activity and total antioxidant activity of the CHS-10LPP reached 70.09% and 82.29%, respectively. Hanani et al. [1] also found that when pomegranate peel powder was added to fish gelatin film, it could obtain up to 71.82% of DPPH radical scavenging activity and 80.02% of total antioxidant activity. The biologically active compounds in LPP, such as anthocyanins and phenolic compounds, are antioxidants with strong antioxidant activity [22]. In addition, it has been reported that polyphenols can trap free radicals by providing phenolic hydrogen [32]. Kalaycıoğlu and Erim [48] indicated that phenolic compounds in pericarp have the ability to chelate metal cations and scavenge free radicals. Therefore, CHS film containing LPP as food packaging has the potential to inhibit food oxidation.

### 3.5. Application as Fresh-Cut Apple Packaging

#### 3.5.1. Physical Appearance

**Figure 7:**
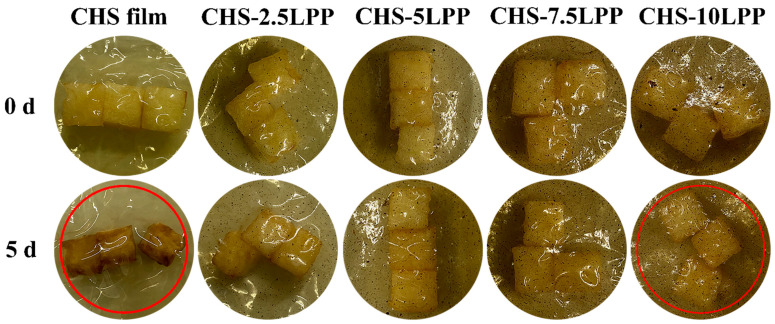
Effect of different films on physical appearance of fresh-cut apples after 5 days storage at 25 °C. The red circles show the comparison between fresh-cut apples packaged in CHS film and CHS-10LPP stored for 5 days.

The digital photos of fresh-cut apple pieces wrapped in different films and stored at room temperature (25 °C) for five days are shown in Figure 7. All apple pieces were white and rich in water before storage. The color of fresh-cut apple packed with CHS film turned black and dark after five days storage. In addition, the shape of fresh-cut apple pieces also shrinks due to water loss. On the contrary, the fresh-cut apple pieces packed with CHS film containing LPP only slightly darkened, and with the increase of LPP content, the shape of fresh-cut apple pieces almost had no visible change. Compared with single-CHS film, CHS-LPP films have lower WS and SD, so it has lower hydrophilicity, which was conducive to the preservation of fresh-cut fruits by maintaining the local high humidity caused by water loss from fruits [49]. The films containing LPP have better barrier properties, which can prevent the water exchange between the external environment and the packaged foods, thus prolonging the shelf life of fresh-cut apple. In addition, the antioxidant capacity of CHS-LPP films was an important factor in inhibiting the browning of fresh-cut apple. Polyphenol oxidase (PPO)-mediated browning is a main cause of color change in fresh-cut apples [50]. Antioxidants are seen to be more potent inhibitors of PPO [51]. Therefore, CHS film containing LPP can effectively inhibit the browning of fresh-cut fruits, thus maintaining a higher appearance quality of fruits.

#### 3.5.2. Weight Loss

**Figure 8:**
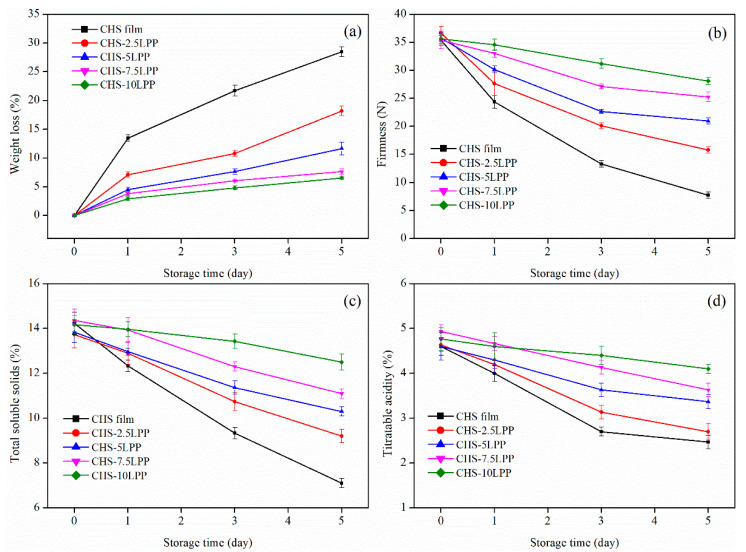
The weight loss (**a**), firmness (**b**), total soluble solids (**c**), and titratable acidity (**d**), of fresh-cut apple packaged in different films during storage. Fruit packaged with different films were stored at 25 °C for 0, 1, 2, 3, 4, and 5 days.

The change in weight loss of fresh-cut apples packaged in different films during storage is shown in Figure 8a. The weight loss of fresh-cut apples in each group showed an increasing trend during storage. The reason for this phenomenon was dry matter consumption and stomatal transpiration caused by respiration [50]. The weight loss rate of fresh-cut apples packaging in CHS film reached about 28% after storage for five days, which was much higher than that of packaged in CHS-LPP groups. In particular, among the fresh-cut apples packaged in CHS-LPP films, the weight loss of fresh-cut apples packaged in CHS-10LPP was the lowest (6.5%). This may be related to the formation of hydrogen bonds between the LPP and the CHS matrix, which improves the water vapor barrier properties of the CHS-LPP film, thereby greatly inhibiting the escape of water vapor from the package.

#### 3.5.3. Firmness

Firmness is the most important physical attribute that affects consumer acceptance [52]. The firmness changes of fresh-cut apples packaged with CHS film and CHS-LPP film during storage are shown in Figure 8b. The firmness of fresh-cut apples packed in CHS film decreased about 78% during five days storage, which was much higher than that of packaged in CHS-LPP groups. With the increase of LPP content in CHS film, the firmness of fresh-cut apple decreased more slowly during storage, and the firmness of fresh-cut apple in CHS-10LPP was the highest at the end of storage period. The main reasons for softening of fruits were the degradation of cell wall caused by fruit respiration, water loss, and damage of structure and tissue by mold [53]. The films containing LPP displayed lower WVP than CHS film, and CHS-LPP film can provide a barrier, improved the antibacterial ability of fruit, reduced water loss and respiration, and thus reduced the loss rate of fruit hardness. In addition, pure CHS film had higher affinity with water and exhibited lower water barrier and therefore failed to form an effective water barrier layer to reduce fruit deterioration [35]. Rojas-Bravo et al. [26] also found that apple slices coated with mango peel/starch coating had a higher firmness after storage for six days, which was related to the increase in penetration resistance of the physical barrier.

#### 3.5.4. Total Soluble Solids

Figure 8c shows the total soluble solids of fresh-cut apple packaged in different films during storage. It can be seen that the total soluble solids content of fresh-cut apple in all packages showed a downward trend during storage. This was due to the reduction of carbohydrates and the breakdown of glycosides into subunits caused by the respiration of fresh-cut apples [52]. The decrease rate of total soluble solids of fresh-cut apple packaged with CHS-LPP film was lower than that of pure CHS film during the whole storage period, indicating that the addition of LPP significantly reduced the conversion rate of reducing sugar of fresh-cut apple. Rojas-Bravo et al. [26] also reported mango peel/starch-coated apple slices maintain higher total soluble solids after storage. CHS-LPP film had a more effective effect on slowing down the metabolism and respiratory activity of fresh-cut apple, which may be related to the interaction between polyphenol-rich LPP and apple cell membrane [50]. Gull et al. [52] found that CHS coating and pomegranate peel extract reduced the respiration rate of apricot fruit and thus reduced the loss rate of total soluble solids.

#### 3.5.5. Titratable Acidity

Figure 8d shows the titratable acidity of fresh-cut apple packaged in different films during storage. The titratable acid content of fresh-cut apples packaged with CHS film decreased by about 46% after storage, while the titratable acid content of fresh-cut apples packaged with CHS-LPP films was higher than that of CHS film after storage, and CHS-10LPP had the highest titratable acid content (4.1%). The reduction in titratable acid was the result of the metabolism in apples or the use of organic acids in respiratory process [35]. The CHS film containing LPP has better barrier property, which weakens the metabolism and respiration of apple, thus reducing the consumption of organic acids. Yang et al. [54] reported that the CHS coating containing blueberry leaf extract maintained high titratable acid of blueberries during storage, which was due to the high barrier properties, limiting the supply of oxygen.

### 3.6. Application for Preserving the Apple Juice

**Figure 9:**
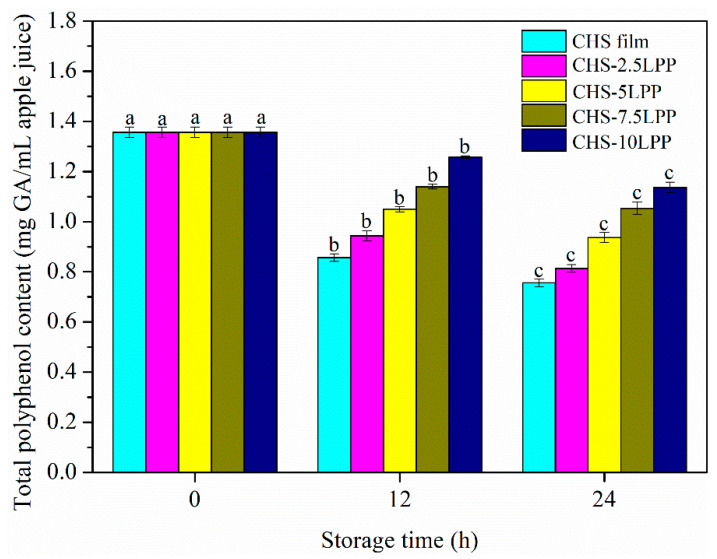
Total phenolic content (mg GA/mL apple juice) of apple juice packaged with different films during storage. Juice packaged with different films were stored at 25 °C for 0, 12, and 24 h. Different lowercase letters within the same packaging indicate significantly different as determined by Duncan’s test (*p* < 0.05).

Figure 9 shows the total polyphenol content of apple juice packaged with different films during storage. During storage, the total polyphenol content of all samples decreased significantly (*p* < 0.05), and the total polyphenol content of apple juice in CHS film decreased the fastest. In CHS-LPP films, with the increase of LPP content, the decrease rate of total polyphenol content became slow. After 24 h, the total polyphenol content of apple juice in CHS-10LPP was the highest. One of the important factors influencing the oxidation of polyphenols is oxygen. The films containing LPP have good barrier properties, which was beneficial to reduce oxygen entering the film. In addition, PPO can catalyze the oxidation of polyphenol compounds into quinones, and antioxidants can effectively inhibit the activity of PPO [51]. Therefore, the bioactive components released by CHS-LPP films have strong antioxidant activity, which can effectively inhibit the oxidation of apple juice. Wu et al. [29] also found that fish gelatin film containing polyphenol compounds can effectively reduce the oxidation of apple juice and prolong the shelf life.

## 4. Conclusions

Active food packaging film was successfully developed by adding LPP into CHS matrix. SEM observed that the incorporation of LPP made the surface and cross-section of the CHS film rougher. FT-IR and XRD results confirmed the formation of intermolecular hydrogen bonds between LPP and CHS. The intermolecular interaction significantly increased the TS of the CHS-LPP films. Due to the hydrophobicity of LPP, CHS-LPP films have higher water resistance than CHS film. LPP contributed to the excellent the UV-vis light barrier and antibacterial and antioxidant properties of the films. Furthermore, CHS-LPP film can significantly inhibit the browning of fresh-cut apple and remarkably lowered the weight loss, firmness, titratable acidity, and total soluble solids of fresh-cut apple during storage. The active packaging film has better preservation effect on apple juice. Therefore, the results of this study indicate that LPP has great potential as an additive for CHS film because the obtained LPP is economically feasible since it is derived from waste. CHS-LPP film is a promising active food packaging material that can prevent food from oxidation and deterioration and thus extend the shelf life of food. Our results suggested CHS-LPP films (especially CHS-10LPP) could be utilized as active packaging materials to prevent food spoilage and oxidation and thus prolong the shelf-life of foods.

## Figures and Tables

**Table 1:** *L*, *a*, *b*, and Δ*E* of the films.

Films	*L*	*a*	*b*	Δ*E*
CHS film	87.47 ± 1.18 ^a^	−2.49 ± 0.09 ^e^	0.13 ± 0.12 ^e^	0.00 ± 0.00 ^e^
CHS-2.5LPP	81.77 ± 0.55 ^b^	−1.47 ± 0.15 ^d^	6.87 ± 0.81 ^d^	8.91 ± 0.51 ^d^
CHS-5LPP	79.31 ± 0.19 ^c^	−0.17 ± 0.12 ^c^	11.83 ± 1.52 ^c^	14.48 ± 1.03 ^c^
CHS-7.5LPP	76.41 ± 0.56 ^d^	1.67 ± 0.23 ^b^	20.59 ± 0.62 ^b^	23.65 ± 0.29 ^b^
CHS-10LPP	74.09 ± 0.66 ^e^	2.91 ± 0.09 ^a^	25.17 ± 1.34 ^a^	28.91 ± 0.77 ^a^

Values are expressed as mean ± standard deviation. Different letters in the same column indicate significantly different (*p* ˂ 0.05).

**Table 2:** Thickness, MC, WS, and SD of films.

Films	Thickness (mm)	MC (%)	WS (%)	SD (%)
CHS film	0.058 ± 0.006 ^e^	31.663 ± 0.272 ^a^	20.487 ± 0.798 ^a^	164.547 ± 3.868 ^a^
CHS-2.5LPP	0.071 ± 0.007 ^d^	26.041 ± 0.869 ^b^	18.341 ± 0.251 ^b^	128.501 ± 2.265 ^b^
CHS-5LPP	0.082 ± 0.003 ^c^	17.107 ± 0.816 ^c^	16.263 ± 0.411 ^c^	100.687 ± 4.642 ^c^
CHS-7.5LPP	0.097 ± 0.009 ^b^	16.583 ± 0.112 ^c^	15.223 ± 0.172 ^d^	73.433 ± 3.365 ^d^
CHS-10LPP	0.115 ± 0.003 ^a^	16.581 ± 0.434 ^c^	14.342 ± 0.251 ^e^	56.971 ± 1.758 ^e^

Values are expressed as mean ± standard deviation. Different letters in the same column indicate significantly different (*p* ˂ 0.05).

**Table 3:** WVP, TS, and EAB of films.

Films	WVP (×10^−7^ g m^−1^ h^−1^ Pa^−1^)	TS (MPa)	EAB (%)	EM (MPa)
CHS film	6.67 ± 0.09 ^a^	10.98 ± 0.56 ^e^	24.79 ± 1.59 ^a^	44.41 ± 2.61 ^e^
CHS-2.5LPP	6.44 ± 0.07 ^b^	12.47 ± 0.11 ^d^	19.85 ± 0.62 ^b^	62.86 ± 1.83 ^d^
CHS-5LPP	6.13 ± 0.11 ^c^	14.28 ± 0.09 ^c^	18.35 ± 0.45 ^c^	77.84 ± 1.14 ^c^
CHS-7.5LPP	5.41 ± 0.09 ^d^	16.06 ± 0.16 ^b^	14.51 ± 0.42 ^d^	110.74 ± 2.46 ^b^
CHS-10LPP	4.67 ± 0.06 ^e^	16.82 ± 0.39 ^a^	9.58 ± 0.63 ^e^	176.25 ± 12.61 ^a^

Values are expressed as mean ± standard deviation. Different letters in the same column indicate significantly different (*p* ˂ 0.05).

**Table 4:** Antioxidant and antibacterial activity of the CHS-LPP films.

Films	Diameter of Inhibition Zone (mm)			
	*Escherichia coli*	*Staphylococcus aureus*	DPPH Radical Scavenging Activity (%)	Total Antioxidant Activity (%)
CHS film	10.81 ± 0.16 ^e^	11.03 ± 0.12 ^e^	-	-
CHS-2.5LPP	11.81 ± 0.06 ^d^	12.04 ± 0.08 ^d^	45.07 ± 1.37 ^d^	54.52 ± 1.24 ^d^
CHS-5LPP	12.45 ± 0.08 ^c^	12.72 ± 0.09 ^c^	55.53 ± 0.99 ^c^	64.47 ± 1.55 ^c^
CHS-7.5LPP	13.18 ± 0.11 ^b^	13.48 ± 0.08 ^b^	65.06 ± 1.36 ^b^	74.41 ± 1.14 ^b^
CHS-10LPP	14.02 ± 0.08 ^a^	14.41 ± 0.09 ^a^	70.09 ± 1.37 ^a^	82.29 ± 1.54 ^a^

Values are expressed as mean ± standard deviation. Different letters in the same column indicate significantly different (*p* ˂ 0.05).

## Data Availability

Not applicable.
